# Superior load‐to‐failure in an all‐suture anchor system for all‐inside meniscal repair compared to a PEEK‐cage anchor system in an experimental cadaveric test setting

**DOI:** 10.1002/jeo2.12110

**Published:** 2024-07-24

**Authors:** Lorenz Pichler, Gyula Kiss, Thomas Sator, Andrea Schuller, Sam A. Kandathil, Marcus Hofbauer, Thomas Koch, Lena Hirtler, Thomas Tiefenboeck

**Affiliations:** ^1^ Department of Orthopaedics and Trauma Surgery Medical University of Vienna Vienna Austria; ^2^ Center for Musculoskeletal Surgery Charité – University Medicine Berlin Berlin Germany; ^3^ Center for Anatomy and Cell Biology, Division of Anatomy Medical University of Vienna Vienna Austria; ^4^ Research Group for Structural Polymers Technical University Vienna Vienna Austria

**Keywords:** all‐suture anchor, arthroscopy, meniscal repair, PEEK‐cage anchor, save the mensicus

## Abstract

**Purpose:**

The purpose of this study was to compare the biomechanical properties of a latest generation all‐suture anchor repair device (ASARD) for meniscal repair with that of a latest generation PEEK‐cage anchor repair device (PCARD) in an experimental setting using cadaveric menisci.

**Methods:**

Twenty‐six menisci were obtained from the knees of fresh body donors. Artificially created meniscal lesions were treated randomly, using a single stitch with either an ASARD or a PCARD. Cyclic biomechanical testing, utilising a universal material testing machine and following an established protocol, was carried out and load‐to‐failure (LTF), displacement, stiffness, and mode‐of‐failure (MOF) reported.

**Results:**

Mean LTF was found to be 61% higher in the ASARD group at 107.10 N (standard deviation [SD], 42.34), compared to 65.86 N (SD, 27.42) in the PCARD group with statistical significance (*p* = 0.022). The ASARD exhibited a trend towards higher stiffness (10.35 N; SD, 3.92 versus 7.78 N; SD; 3.59) and higher displacement at cycles one, 100, and 499 (1.64, 3.27, and 4.17 mm versus 0.93, 2.19, and 2.83 mm) compared to the PCARD. Cheese wiring was the most common mode‐of‐failure in both groups (76.9%).

**Conclusions:**

This study demonstrates that an ASARD shows a higher mean LTF than a PCARD when compared in an experimental biomechanical setting.

**Level of evidence:**

Level III

**Abbreviations**
ASARD: all‐suture anchor repair device
LTF: load‐to‐failure
MOF: mode‐of‐failure
PCARD: PEEK‐cage anchor repair device
PEEK: polyether ether ketone
SD: standard deviation

## INTRODUCTION

Meniscal injuries are among the most common musculoskeletal injuries and meniscectomy has been shown to result in higher rates of osteoarthritis when compared to meniscal repair [6, 9, 14, 17, 20]. Consequently, the number of repair procedures carried out increased, and their cost‐effectiveness was proven, leading to the momentum behind the phrase ‘Save the Meniscus’ [1, 3, 13, 18]. To date, several generations of meniscal repair devices (MRDs) exist. To minimise the anchor diameter and potentially reduce soft tissue irritation, some manufacturers of these devices replaced the rigid anchor used for peripheral fixation with an anchor made of suture material thus naming them ‘all‐suture anchor repair device’ (ASARD) [15, 22]. However, evidence on the biomechanical properties of ASARDs compared to non‐suture anchor devices is low. This study compared the biomechanical properties of an ASARD with those of a PEEK‐cage anchor repair device (PCARD), both representing the latest generation of their kind. The hypothesis formulated was that the ASARD demonstrates a higher load‐to‐failure (LTF) when compared to the PCARD in an experimental, biomechanical setting, utilising cadaveric menisci and an established biomechanical test protocol.

## METHODS

The study protocol was approved by the local ethics committee of (EK‐Nr. 1656/2021) and the study was conducted in accordance with the Declaration of Helsinki. Written informed consent was obtained pre‐mortem from all body donors by the center for anatomy from which menisci were obtained.

**Figure 1:**
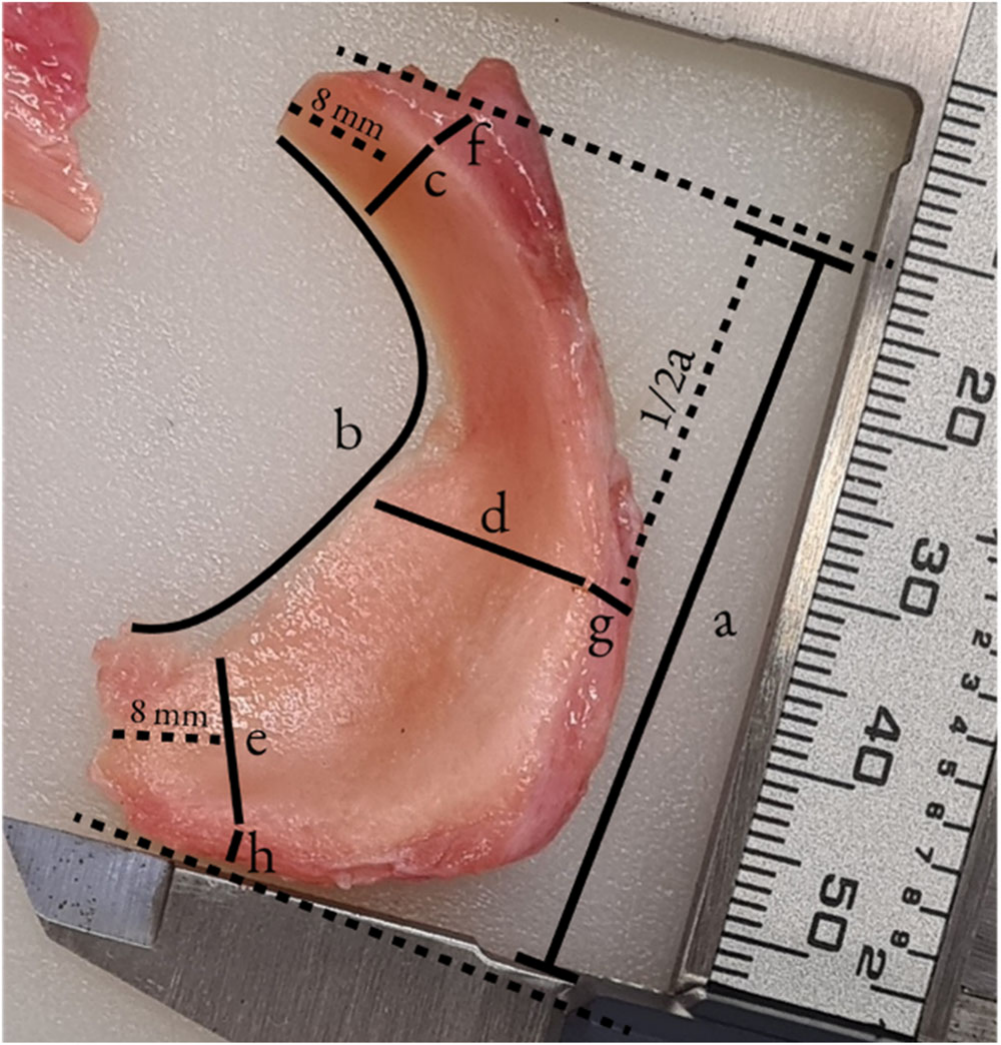
Meniscus measurements.

Menisci were obtained from injury‐free knees, of fresh body donors through anterior arthrotomy, and dissection of the menisci at their anterior and posterior roots by an orthopaedic surgery resident. Obtained menisci were subjected to visual inspection for lesions and degeneration. Body donor demographics and mean meniscus size measured as illustrated in Figure 1 are reported according to sex and device group in Tables 1 and 2.

**Table 1:** Body donor demographics.

		Overall	ASARD	PCARD
Donor age, years, mean		81.8 (range; 68–98)	80.0 (range; 75–88)	81.0 (range; 68–98)
Donor sex	Female	17 (65.4%)	9 (69.2%)	8 (61.5%)
	Male	9 (34.6%)	4 (30.8%)	5 (38.5%)
Meniscus side	Lateral	13 (50.0%)	6 (46.1%)	7 (53.8%)
	Medial	13 (50.0%)	7 (53.8%)	6 (46.1%)

Abbreviations: ASARD, all‐suture anchor repair device; PCARD, PEEK‐cage anchor repair device.

**Table 2:** Meniscus size.

	Overall	Male	Female	Lateral	Medial	ASARD	PCARD
Anterior‐posterior diameter (a)	39.65 ± 5.68	41.60 ± 5.83	38.62 ± 5.50	37.35 ± 5.37	41.95 ± 5.19	39.07 ± 5.43	40.24 ± 2.36
Inner circumference (b)	55.72 ± 8.36	59.17 ± 10.36	53.90 ± 6.74	55.00 ± 11.03	56.45 ± 4.78	55.84 ± 8.88	55.61 ± 7.47
Radius							
Anterior horn (c)	10.27 ± 1.97	10.05 ± 1.72	10.39 ± 2.13	11.20 ± 1.94	9.34 ± 1.56	10.26 ± 1.37	10.28 ± 2.36
Pars intermedia (d)	8.81 ± 1.33	8.99 ± 1.14	8.72 ± 1.44	9.61 ± 0.90	8.01 ± 1.22	8.64 ± 1.15	8.98 ± 1.42
Posterior horn (e)	12.30 ± 2.30	13.15 ± 2.65	11.85 ± 2.02	11.17 ± 1.91	13.42 ± 2.15	12.31 ± 2.23	12.30 ± 2.27
Thickness							
Anterior horn (f)	4.94 ± 0.93	5.34 ± 0.83	4.72 ± 0.93	4.84 ± 0.79	5.03 ± 1.08	4.63 ± 0.64	5.24 ± 1.04
Pars intermedia (g)	5.40 ± 1.43	5.21 ± 1.04	5.50 ± 1.61	5.49 ± 1.05	5.31 ± 1.77	5.01 ± 1.54	5.78 ± 1.11
Posterior horn (h)	5.27 ± 1.01	5.18 ± 1.21	5.31 ± 0.92	5.15 ± 1.24	5.38 ± 0.74	5.07 ± 1.06	5.46 ± 0.87

*Note*: Measurements in millimetres (mm). Values represent means unless stated otherwise ± standard deviation.

Abbreviations: ASARD, all‐suture anchor repair device; PCARD, PEEK‐cage anchor repair device.

**Figure 2:**
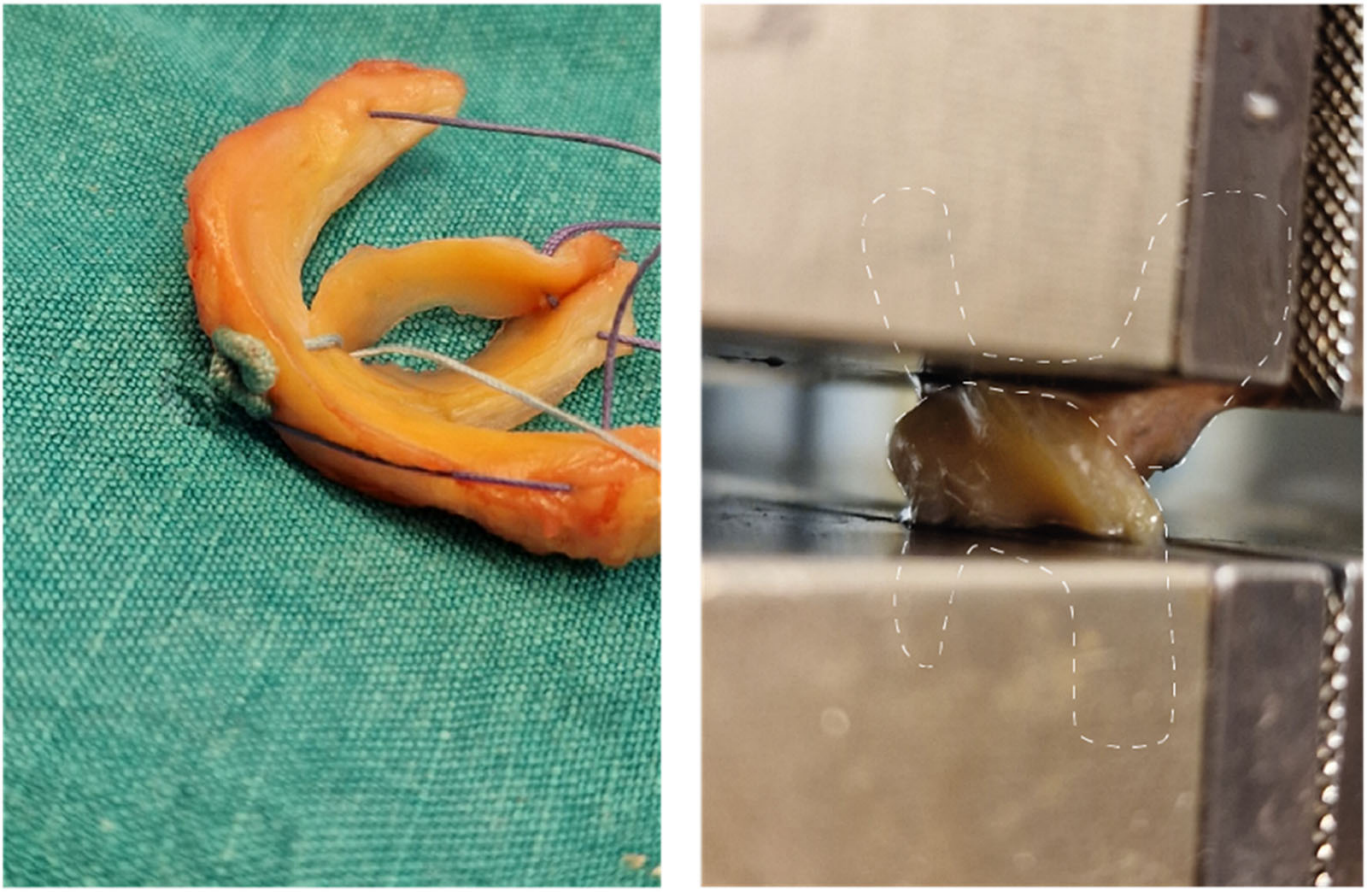
Meniscus preparation with all‐suture anchor (left). Setup for biomechanical testing, dashed lines depict the outlines of the inner and outer part of the meniscus (right).

Following dissection, menisci were subjected to preparation and testing. An artificial meniscal lesion was created through a 10 mm longitudinal incision to the pars intermedia at 3 mm measured from the outer rim of the meniscus, using an 11‐blade scalpel. Menisci were randomly assigned for single‐stitch repair by a specialist for orthopaedics surgery (T.T.) using either an ASARD (FiberStitch™; Arthrex) or PCARD (FAST‐FIX™ Flex; Smith & Nephew). After the repair, the lesions were extended along the longitudinal fibres, reaching both the anterior and posterior horns. This process created two segments of the meniscus, an inner and an outer part, which remained connected solely by the stitches applied. The inner part of the anterior horn was then connected to the inner part of the posterior horn and the outer part of the anterior horn to the outer part of the anterior horn by a 3‐0 Vicryl suture (Ethicon Inc.), resulting in a figure‐of‐eight configuration (Figure 2).

After preparation, testing was carried out by two residents (G.K. & L.P) and a specialist (T.T.) for orthopaedics surgery at a laboratory for mechanical engineering under the guidance of a senior scientist with more than two decades of experience in material testing (T.K.). To ensure comparability the testing protocol applied and the parameters recorded closely followed the protocol for the testing of meniscal repair devices established by Bachmaier et al. [2]. A calibrated universal material testing machine (Zwick Z050; ZwickRoell) equipped with a force sensor was used. The inner and outer parts of the menisci were secured inside the machine using vertically aligned clamps as display in Figure 2 and care was taken to avoid clamping the applied stitches. Machine data output was recorded using ZwickRoell firmware.

**Figure 3:**
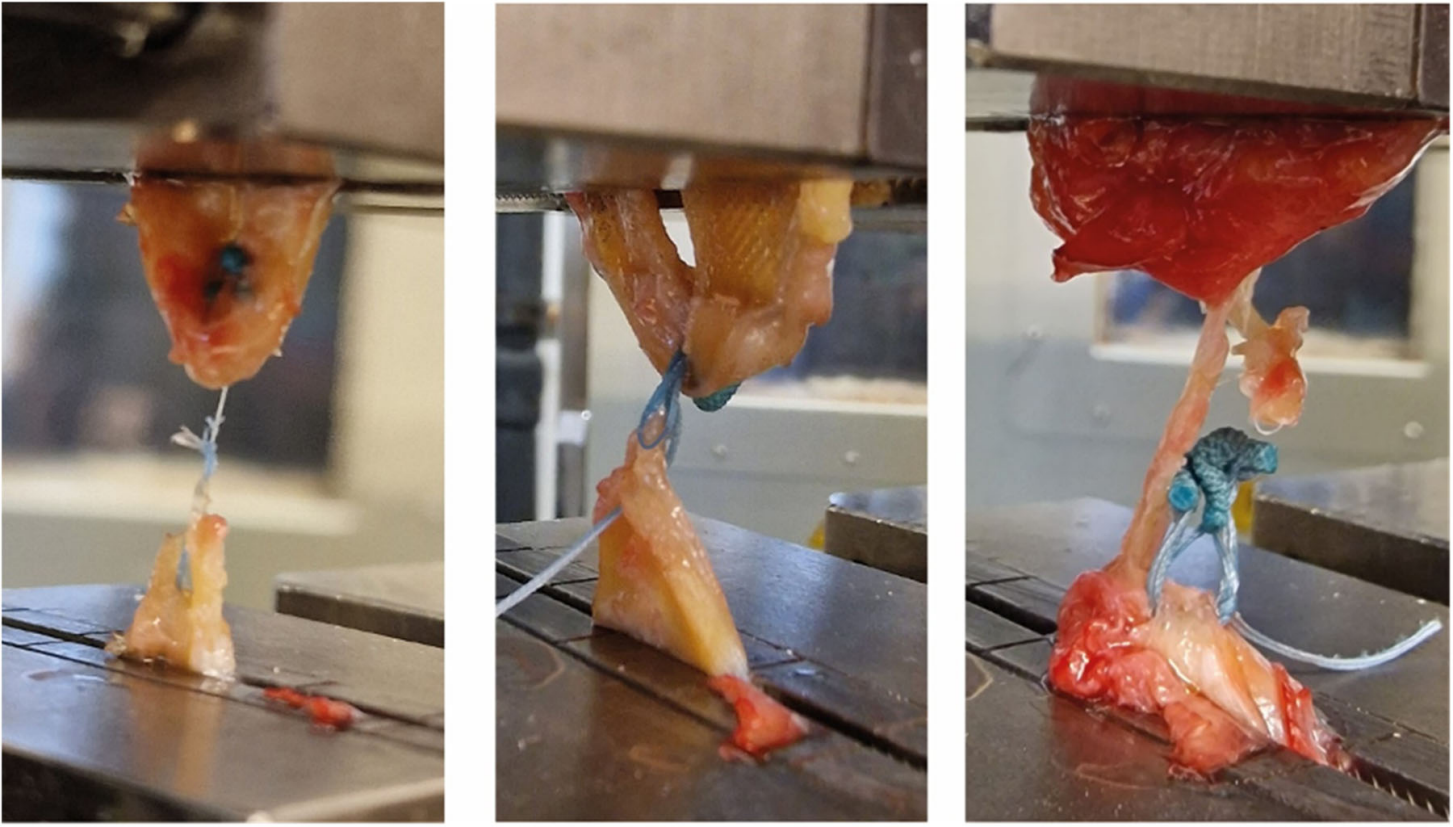
Modes of failure: thread rupture (left), cheese wiring (middle), and anchor slippage (right).

Testing began with the application of a 20 N preload to the meniscus. Following this, cyclic testing was initiated. Following preloading, 500 cycles of relaxation to 5 N and consecutive re‐tensioning to 20 N at a frequency of 1 Hz took place. Displacement between the two parts of the menisci defined as the increase of distance between clamps gained at each cycle of tensioning was recorded at cycles 1, 100, and 499 through load‐displacement curves. Following 500 cycles, load to failure was determined by gradually increasing the pulling force at a rate of 12.5 mm/s. The load‐to‐failure (LTF) was defined as the force applied at the moment at which loss of resistance was recorded. The mode‐of‐failure (MOF) was categorised as either suture rupture, anchor slippage, or cheese wiring as illustrated in Figure 3 and reported by two members of the team independently. Device stiffness (N/mm) was defined as LTF divided by the displacement recorded at failure.

### Statistical analysis

To ensure methodological quality, the study protocol adhered to the guidelines outlined in the QUACS checklist, specifically designed for observational cadaveric studies [23]. Descriptive analyses of body donor demographics and meniscal size measurements were carried out, and mean, median, and standard deviation reported wherever applicable. A post hoc power analysis was carried out using G*Power (version 3.1.9.6 [10]) and statistical power was found at 0.5 (two‐tailed *t*‐test, effect size 0.8, alpha error probability 0.05). Normal distribution was assessed using Shapiro–Wilk test, and differences in means were evaluated accordingly, using either Mann–Whitney *U*, Fisher's Exact, or students *t*‐tests. Data were analysed using SPSS (version 24.0, SPSS Inc.).

## RESULTS

**Figure 4:**
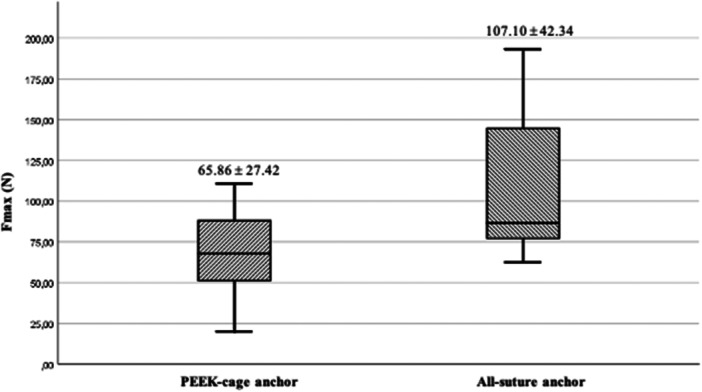
Box plots with mean load to failure compared between groups, *p* = 0.022.

Following the exclusion of two menisci bearing signs of lesions at initial inspection, a total of 26 menisci were included. No statistically significant differences were found for body donor age, distribution of sex, and distribution of meniscus side between groups. The mean LTF for the ASARD group was reported significantly higher at 107.10 N (SD, 42.34) as compared to the PCARD group at 65.68 N (SD, 27.42; *p* = 0.022*; *r* = 0.44; Table 3; Figure 4). Regarding MOF unanimous reports were made by the observers in all cases. Cheese wiring was identified as MOF in 20 cases (76.9%; ASARD 10 cases; PCARD 10 cases), suture rupture in five cases (19.2%; ASARD 2 cases; PCARD 3 cases), and anchor slippage in one case (3.8%, ASARD). Displacement measurements at cycles one, 100, and 499 showed that the ASARD group experienced displacements of 1.34 mm (SD: 1.29), 2.75 mm (SD: 2.41), and 3.53 mm (SD: 3.00), respectively. In comparison, the PCARD group's displacements were slightly higher at 1.64 mm (SD: 1.36; *p* = 0.072), 3.27 mm (SD: 2.04; *p* = 0.11), and 4.17 mm (SD: 2.33; *p* = 0.168), respectively. At failure, the ASARD group's menisci demonstrated a stiffness of 10.35 N/mm (SD: 3.92), compared with the PCARD group's stiffness of 7.78 N/mm (SD: 3.59; *p* = 0.94).

**Table 3:** Load to failure, displacement, stiffness, and mode of failure.

	Overall	All‐suture anchor	PEEK‐cage anchor	*p*‐Value
Load to failure				
Mean	86.48 ± 40.78	107.10 ± 42.34	65.86 ± 27.42	0.022*
Median	79.32	86.61	67.78	
Minimum	19.98	62.51	19.98	
Maximum	193.27	193.27	110.75	
Displacement				
Round 1, mean	1.34 ± 1.29	1.64 ± 1.36	0.93 ± 1.18	n.s.
Round 100, mean	2.75 ± 2.41	3.27 ± 2.04	2.19 ± 2.73	n.s.
Round 499, mean	3.53 ± 3.00	4.17 ± 2.33	2.83 ± 3.57	n.s.
Stiffness				
Mean	9.06 ± 3.91	10.35 ± 3.92	7.78 ± 3.59	n.s.
Median	8.81	9.49	8.12	
Minimum	2.70	5.71	2.70	
Maximum	19.11	19.11	14.56	
Mode of failure				
Thread rupture		2	3	
Cheese‐wiring		10	10	
Anchor slippage		1	0	

*Note*: Measurements in Newton (N). All values represent means unless stated otherwise ± mean standard deviation; n.s., not significant.

## DISCUSSION

The main finding of this study is that an ASARD exhibits a higher LTF when compared to a PCARD in an experimental cadaveric setting. Therefore, its hypothesis was accepted. More specifically, the mean LTF demonstrated by the ASARD evaluated in this study was 61% higher than that of the PCARD. These findings are consistent with those of earlier generations of MRDs tested in the study by Bachmaier et al. who compared an ASARD to a different PCARD [2].

Notably, the mean LTF reported for the ASARD by Bachmaier et al. exceeds both the findings of this study and the manufacturer's reported values (145, 107, and 69 N, respectively). While testing protocols were nearly identical, variations in the methods employed for the tensioning of the applied stitch were found. Bachmaier et al utilised a spring‐loaded tensiometer set to 50 N. In contrast, in the presented study and in the manufacturer's own assessment [7], stitches were tensioned manually according to the surgical guidelines of the ASARD. Currently, there is a lack of evidence regarding the tension force that can be manually applied to the stitches of an all‐inside MRD, as well as the variability of this force. Consequently, the impact of a standardised tensioning force on the overall LTF of such devices remains unclear and should be subject of further investigations.

Biomechanical evaluations of MRDs often focus on LTF as the determinant of success and superiority of devices, though very little evidence exists on the in‐vivo LTF such devices have to withstand. Kirsch et al. in their early investigations on the in vivo forces acting on meniscal sutures reported them to never exceed 10 N throughout knee flexion up to 114° [11]. However, the extent to which weight bearing increases these forces and whether there is a need for more LTF than what modern all‐inside MRDs currently provide remains uncertain.

The ASARD tested in this study exhibited its higher LTF while also showing a trend towards higher ultimate stiffness when compared to the PCARD. Higher stiffness in MRDs might be conducive to tissue healing [4, 5] but was also associated with an increased risk of cheese‐wiring [16]. Although the sample size of this study was insufficient to test for significant differences in MOF between devices, it raises the question of whether maximum stiffness in MRDs is beneficial, or if a certain degree of flexibility might offer biomechanical advantages.

Of note, the ASARD achieved higher LTF and stiffness but also showed a tendency for higher displacement amongst all cycles of testing when compared to the PCARD. Whether the higher displacement found is a result of increased LTF and stiffness and how these parameters translate to patient outcome remains unclear. Considering failure rates as high as 11.9% in all‐inside meniscal repairs [19], there is a critical need for further research to understand how to harmonise the biomechanical parameters of MRDs to optimise in‐vivo performance. Ultimately, MRDs have to be compared with respect to their clinical and patient‐reported outcomes.

The experimental nature of this study bears several limitations. While the sample size of the presented study was limited by the number of lesion‐free menisci, it was comparable to that of similar studies [8, 12, 21]. Cadaveric studies inherently depict a time‐zero scenario, leaving aside the natural healing process and the ingrowth of implants and sutures. Furthermore, the test setting applied represents a very simplified abstraction of forces acting on MRDs in the knee joint and the age of the body donors in this study may not be representative of a typical patient requiring meniscal repair. However, such simplified biomechanical test settings are essential to identify parameters with a potential impact on the real‐life performance of implants and should be followed by in vivo investigations.

If verified by in vivo investigations, the findings of this study could significantly contribute to efforts aimed at ‘saving the meniscus’ and preventing osteoarthritis.

## CONCLUSION

In a controlled cadaveric test setting, an all‐suture anchor repair device designed for all‐inside meniscal repairs demonstrated superior load‐to‐failure capabilities compared to a PEEK‐cage meniscal repair device. To optimise the design of meniscal repair devices for enhanced in‐vivo performance, further studies are essential to understand how various biomechanical properties interact within these devices.

## AUTHOR CONTRIBUTIONS

All authors contributed to the study conception and design. Material preparation, data collection and analysis were performed by Gyula Kiss, Thomas Tiefenboeck and Lorenz Pichler. The first draft of the manuscript was written by Lorenz Pichler and all authors commented on previous versions of the manuscript. All authors read and approved the final manuscript.

## CONFLICT OF INTEREST STATEMENT

The authors declare no conflict of interest.

## ETHICS STATEMENT

The study protocol was approved by the local ethics committee (EK‐Nr. 1656/2021), and the study was conducted in accordance with the Declaration of Helsinki.

## Data Availability

The data that support the findings of this study are available from the corresponding author upon reasonable request.
